# Engineering of a Biomimetic Pericyte-Covered 3D Microvascular Network

**DOI:** 10.1371/journal.pone.0133880

**Published:** 2015-07-23

**Authors:** Jaerim Kim, Minhwan Chung, Sudong Kim, Dong Hyun Jo, Jeong Hun Kim, Noo Li Jeon

**Affiliations:** 1 Division of WCU Multiscale Mechanical Design, School of Mechanical and Aerospace Engineering, Seoul National University, Seoul, Republic of Korea; 2 School of Mechanical and Aerospace Engineering, Seoul National University, Seoul, Republic of Korea; 3 Fight against Angiogenesis-Related Blindness (FARB) Laboratory, Clinical Research Institute, Seoul National University Hospital, Seoul, Republic of Korea; 4 Department of Biomedical Sciences and Protein Metabolism Medical Research Center, College of Medicine, Seoul National University, Seoul, Republic of Korea; 5 Department of Ophthalmology, College of Medicine, Seoul National University, Seoul, Republic of Korea; 6 Institute of Advanced Machinery and Design, Seoul National University, Seoul, Republic of Korea; Seoul National University, KOREA, REPUBLIC OF

## Abstract

Pericytes enveloping the endothelium play an important role in the physiology and pathology of microvessels, especially in vessel maturation and stabilization. However, our understanding of fundamental pericyte biology is limited by the lack of a robust *in vitro* model system that allows researchers to evaluate the interactions among multiple cell types in perfusable blood vessels. The present work describes a microfluidic platform that can be used to investigate interactions between pericytes and endothelial cells (ECs) during the sprouting, growth, and maturation steps of neovessel formation. A mixture of ECs and pericytes was attached to the side of a pre-patterned three dimensional fibrin matrix and allowed to sprout across the matrix. The effects of intact coverage and EC maturation by the pericytes on the perfused EC network were confirmed using a confocal microscope. Compared with EC monoculture conditions, EC-pericyte co-cultured vessels showed a significant reduction in diameter, increased numbers of junctions and branches and decreased permeability. In response to biochemical factors, ECs and pericytes in the platform showed the similar features with previous reports from *in vivo* experiments, thus reflect various pathophysiological conditions of *in vivo* microvessels. Taken together, these results support the physiological relevancy of our three-dimensional microfluidic culture system but also that the system can be used to screen drug effect on EC-pericyte biology.

## Introduction

Neovascularization is a multistep process beginning with initial vascular angiogenic sprouting followed by migration and association with pericytes and smooth muscle cells [1, 2]. Specifically, pericytes play an important role in the regulation of capillary diameter, tight and adherens junctions, and extracellular matrix protein secretion through interaction with the endothelium [3, 4]. Moreover, abnormal pericyte integration into the blood vessel wall during pathological situations can lead to endothelial hyperplasia and vascular leakage [5]. Above all, diabetic retinopathy and cancer angiogenesis are closely related to aberrations in endothelial cell (EC)-pericyte interactions [6–11]. Thus, due to their important roles in physiological and pathological conditions, EC-pericyte interactions have continued to increase interest.

Despite the importance of pericytes, most *in vitro* studies have focused on generating vascular networks by culturing ECs alone; thus, there are considerable differences regarding *in vivo* blood vessels in terms of vessel morphology and functions [12–14]. Recently, to better mimic *in vivo* vascular systems, perivascular cells and ECs have been cultured together [15–19]. Microfluidic-based systems have been introduced to overcome the limitations associated with conventional large-scale cultures, such as a lack of perfusion and three dimensionality [20–24]. In some studies, ECs and pericytes were injected with hydrogel in microfluidic assays, and cells were grown along the contours of the simple branch-shaped channels [21, 22]. More recently, Jeon *et al*. generated MSC-enveloped vascular networks using growth factors such as VEGF, Ang-1, and TGF-beta via a vasculogenic process [23]. However, multistep neovessel formation associated with pericytes could not be recapitulated in these *in vitro* models, since the features of vascular networks are fixed by channel structures [21, 22] or vessel formation relies on vasculogenic processes [23].

In this study, we engineered physiologically relevant *in vitro* vascular networks that recapitulated the physical interaction between EC and pericyte, as well as the development process of neovascularization. We demonstrated that this microfluidic model can be used as a reliable experimental platform to form a perfusable vessel network derived from the co-culture of multiple cell types.

## Materials and Methods

### Microfluidic device fabrication

Microfluidic devices were fabricated using the replica molding method. Briefly, polydimethylsiloxane (PDMS, Sylgard 184, Dow Corning) elastomer and curing agent were mixed at a ratio of 10:1 (w/w) and poured onto the master mold fabricated by photolithography. The height of the micro channels was 100 μm. After hardening and peeling off from the master mold, four media reservoirs were punched out of the PDMS using a 6-mm biopsy punch, and four small holes for the hydrogel injection port were punctured using a sharpened hypodermic needle (18G). The device was then cleaned with adhesive tape and covalently bonded to the coverslip by treating with an air plasma for 1 minute.

### Cell culture

Human umbilical vein endothelial cells (HUVECs, Lonza, Switzerland) were cultured in endothelial growth medium (EGM-2, Lonza, Switzerland) with full supplements and were used at passage 4. Dermal fibroblasts (DFs, CEFO, Korea) and normal human lung fibroblasts (LFs, Lonza, Switzerland) were grown in fibroblast growth medium (FGM-2, Lonza, Switzerland) with full supplements and were used at passage 6–8. Human placental pericytes (hPC-PL, Promocell, Germany) were cultured in pericyte growth medium (PGM, Promocell, Germany) and used at passage 6–8. All cells were cultured in a humidified 5% CO_2_ incubator at 37°C.

### Angiogenesis assay

Fibronogen (10 mg/ml, F8630, Sigma-Aldrich, Korea) with 0.45 U/ml aprotinin (A1153, Sigma-Aldrich, Korea) solution was prepared in phosphate-buffered saline (Hyclone, USA). Fibroblasts that had detached from the culture plate were centrifuged and resuspended at a concentration of 10^7^ cells/ml. The fibrinogen solution and the cell suspension were mixed at a ratio of 1:3 to yield a final fibrinogen concentration of 2.5 mg/ml. The mixture was then injected into the side channel as soon as it was mixed with thrombin (T4648, 1 U/ml final concentration, Sigma-Aldrich, Korea). After incubating for 30 minutes to allow for fibrin crosslinking, 2.5 mg/ml fibrinogen (with 0.15 U/ml aprotinin) mixed with thrombin (0.5 U/ml) was injected into the central vessel channel. After 2–3 minutes, the upper reservoirs in each device were filled with culture media (EGM-2) and aspirated gently from the lower reservoirs to wet the hydrophobic media channel. The device was incubated at 37°C overnight to dissipate air bubbles that formed between gel-media interface. HUVECs were then suspended at a concentration of 6×10^6^ cells/ml, or for co-culture with pericytes, HUVECs and pericytes were mixed at a ratio of 5:1 (5×10^6^ cells/ml HUVECs, 1×10^6^ cells/ml pericytes). The media in each reservoir were aspirated, and 5 μl of the cell suspension were added to the media channel contralateral to the fibroblast channel. The device was then tilted to 90 degrees in an incubator to attach cell mixtures to the gel-media interface, followed by a 30-minute incubation. All reservoirs were then filled with media and incubated.

### Immunostaining

Samples were fixed with 4% paraformaldehyde (Thermo, USA) for 15 minutes followed by permeabilization with 0.15% triton-X 100 (Sigma-Aldrich, Korea) for 20 minutes at room temperature. Samples were then treated with 3% bovine serum albumin (BSA, Sigma-Aldrich, Korea) for 1 hour at room temperature to minimize nonspecific binding of antibodies. After washing with PBS, samples were incubated overnight with Alexa Fluor 647-conjugated mouse CD31 antibody (monoclonal, Cat. No. 303112, diluted 1:200, Biolegend, USA) as an endothelial cell marker and Alexa Fluor 488-conjugated mouse α-SMA antibody (monoclonal, Cat. No. IC1420G, diluted 1:200, R&D systems, USA) as a pericyte marker. For nuclear staining, samples were incubated for 2 hours with Hoechst 33342 (Cat. No. C10339, diluted 1:1000, Invitrogen, USA). For basement membrane staining, laminin (Cat. No. ab11575, diluted 1:150, Abcam, USA) or collagen IV (Cat. No. ab6568, diluted 1:150, Abcam) primary antibodies were added to the samples overnight at 4°C. After washing three times with PBS, secondary antibodies (Cat. No. A11036, Alexa Fluor 568 Goat Anti-Rabbit IgG, Molecular Probes, USA) were added for 2 hours at room temperature. Samples were then washed three times and maintained in PBS at 4°C until observation.

### Imaging

The 3D reconstructions and cross sections of the vessels were determined using a confocal microscope (Olympus FV1000). All images were captured using a 20× objective lens. To observe the permeability of the vessels, an inverted epifluorescence microscope (Olympus IX81) was used.

### Image analysis

Images were analysed by Fiji (http://fiji.sc/Fiji). Since z-projection of the stack could result in errors in the branch and junction numbers, especially when two vessels are in skew position, the sample stacks were divided into lower and upper parts before z-projection. The projected images were converted into two-dimensional binary images. Gaussian blur and despeckle were used to perform image smoothing and reduce background noises. Each projection image was then converted into an 8-bit binary image by thresholding in accordance with its original three-dimensional image.

The numbers of junctions and branches were counted using the Skeletonize plugin in Fiji. Vessel widths were measured along the two lines that vertically divide the vessel network into three equal parts. Since the images were binary, we could automatically determine the coordination of the points composing the boundary of each vessel. Along the each vertical line, the points that changed the value from 0 to 255 or reverse were picked using the Processing 2 program (http://processing.org/). Vessel width could then be calculated by simply subtracting each y value of the detected points.

### Permeability visualization

Permeability could be visualized by introducing 4 kDa or 70 kDa FITC-dextran (Sigma Aldrich, Korea) into the entrance of the microvasculature. The media in one of the four reservoirs was exchanged with FITC-dextran (5 mM of the 4 kDa and 0.1 mM of the 70 kDa molecule) containing media. When the vessels were sufficiently perfused, FITC-dextran could flow in and escape from the intravascular to extravascular region over time. Sequential images were obtained using IX81 inverted microscope every 20 seconds. Permeability differences between EC monoculture and EC-pericyte co-culture conditions could be compared qualitatively based on the fluorescent intensity of the extravascular region.

### Biochemical factor treatment

VEGF-A (R&D systems, USA), TNF-α and IL-1 α (PeproTech, USA)were reconstituted as an instructor’s manual and diluted at a stated concentration in the media before use. Abovementioned angiogenesis assay were treated with the factors on day 2, and the media were changed every two days. The samples were fixed and imaged at day 4,

### Statistical analysis

Data were shown as means ± standard errors. Statistical significance was defined as p < 0.001 and evaluated using two-tailed, unpaired Student’s t-tests. All data were analysed using Sigma plot 10.0.

### Ethics Statement

HUVECs and LFs were purchased from Lonza, who provided the following ethics statement: ‘These cells were isolated from donated human tissue after obtaining permission for their use in research applications by informed consent or legal authorization.’ DFs isolated from human foreskin, were purchased from CEFO, who provided certification of permission for use of human tissue in research applications by legal authorization. No further permission was required since we used commercially provided human cells in accordance with internal regulation of Seoul National University institutional review board (SNUIRB).

## Results

### A microfluidic platform supports the establishment of pericyte-covered blood vessels via angiogenic processes *in vitro*

**Fig 1:**
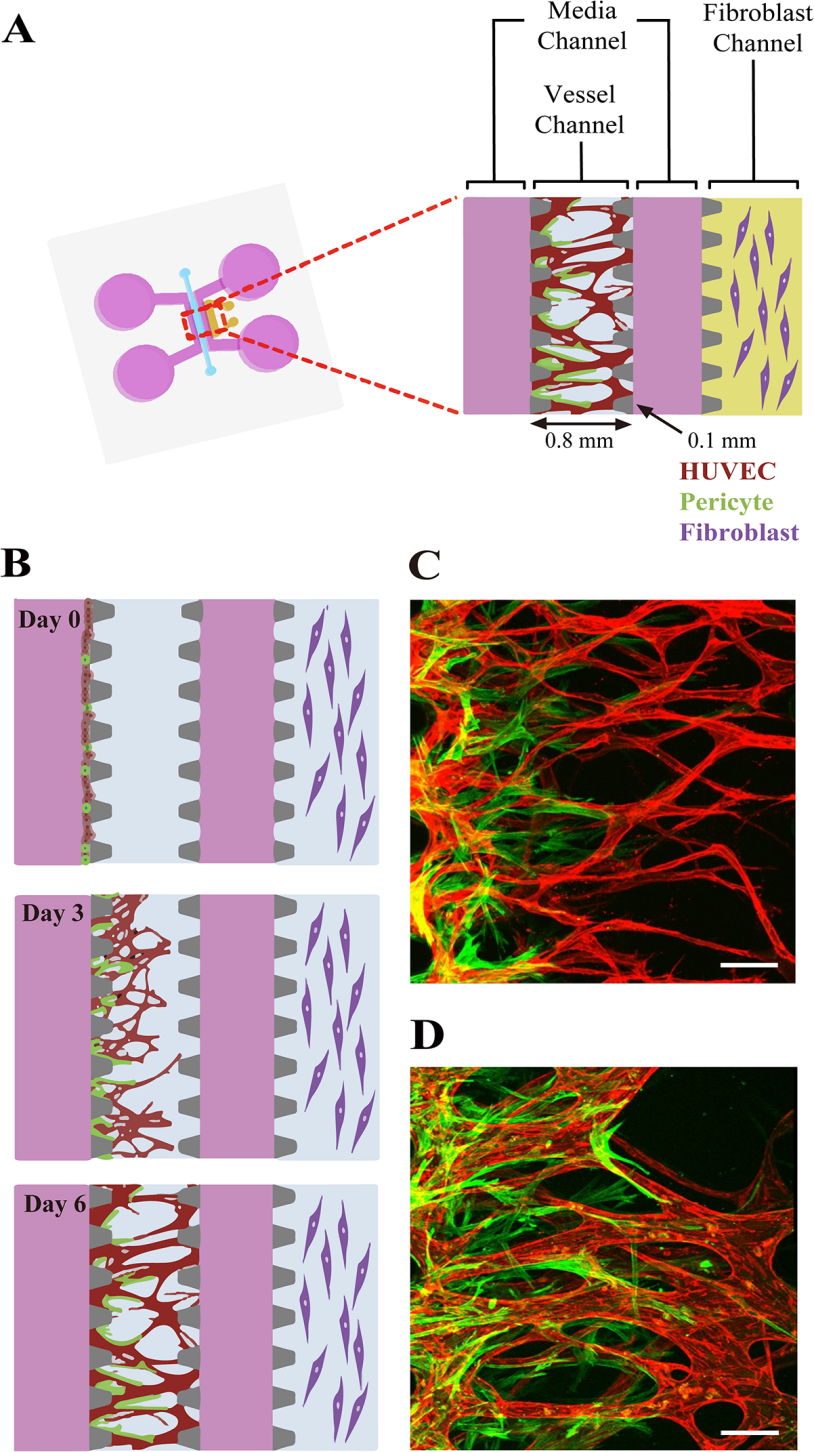
Schematic of the microfluidic system used to mimic the stepwise endothelial-pericyte interaction. (A) The microfluidic device is composed of a central vessel channel, two adjoining media channels, and the outermost fibroblast channel. The vascular network covered by the pericytes was formed in the central channel with assistance from the lateral fibroblasts. (B) The experimental scheme of the stepwise angiogenic process. ECs and pericytes were mixed and attached to the left side of the vessel channel. ECs sprout through the fibrin gel to establish a blood vessel network, and pericytes follow behind the vessel. (C, D) Confocal images show EC patterning prior to pericyte association during the first 3 days (C), and matured pericytes covered the perfusable EC network on day 6 (D). Scale bars, 100 μm.

To mimic *in vivo* neovessel formation followed by pericyte coverage, we adapted our previously described microfluidic platform [12]. Briefly, the microfluidic device is composed of a central vessel channel, two adjoining media channels, and the outermost fibroblast channel (Fig 1A). A mixture of ECs and pericytes was attached to one side of the central acellular fibrin matrix. In response to the directional gradient of biochemical factors secreted by fibroblasts, leading cells of EC sprouts grew toward the opposite end of the central channel and spontaneously formed vacuoles that merged into the intracellular lumen (Fig 1B and 1C). After 5 or 6 days of culture, when the leading portion of the angiogenic sprouts reached the end of the fibrin matrix, ECs robustly developed interconnecting and perfusable vascular networks with tightly adhered pericytes on the basolateral surface of the blood vessels. (Fig 1B and 1D).

We examined two different primary fibroblast strains, each derived from human lung and dermis, the cell types of which support morphogenesis of endothelial cells via paracrine interactions [12, 24, 25]. In both cases, ECs showed robust angiogenic sprouting in response to fibroblast-derived factors to form interconnected vessel networks within 6 days of co-culture. In terms of the angiogenic activity of ECs, LFs more potently induced sprout outgrowth and vascularization of the central channel of the device, as shown based on the relatively denser vascular networks formed in the LF co-culture experiments. However, co-culture with LFs failed to mimic the close association between ECs and pericytes (S1A and S1C Fig), whereas co-culture with DFs could successfully establish adjacent contacts between the two cell types (S1B and S1D Fig).

### Intact coverage of the vascular network by surrounding pericytes promotes vessel maturation

**Fig 2:**
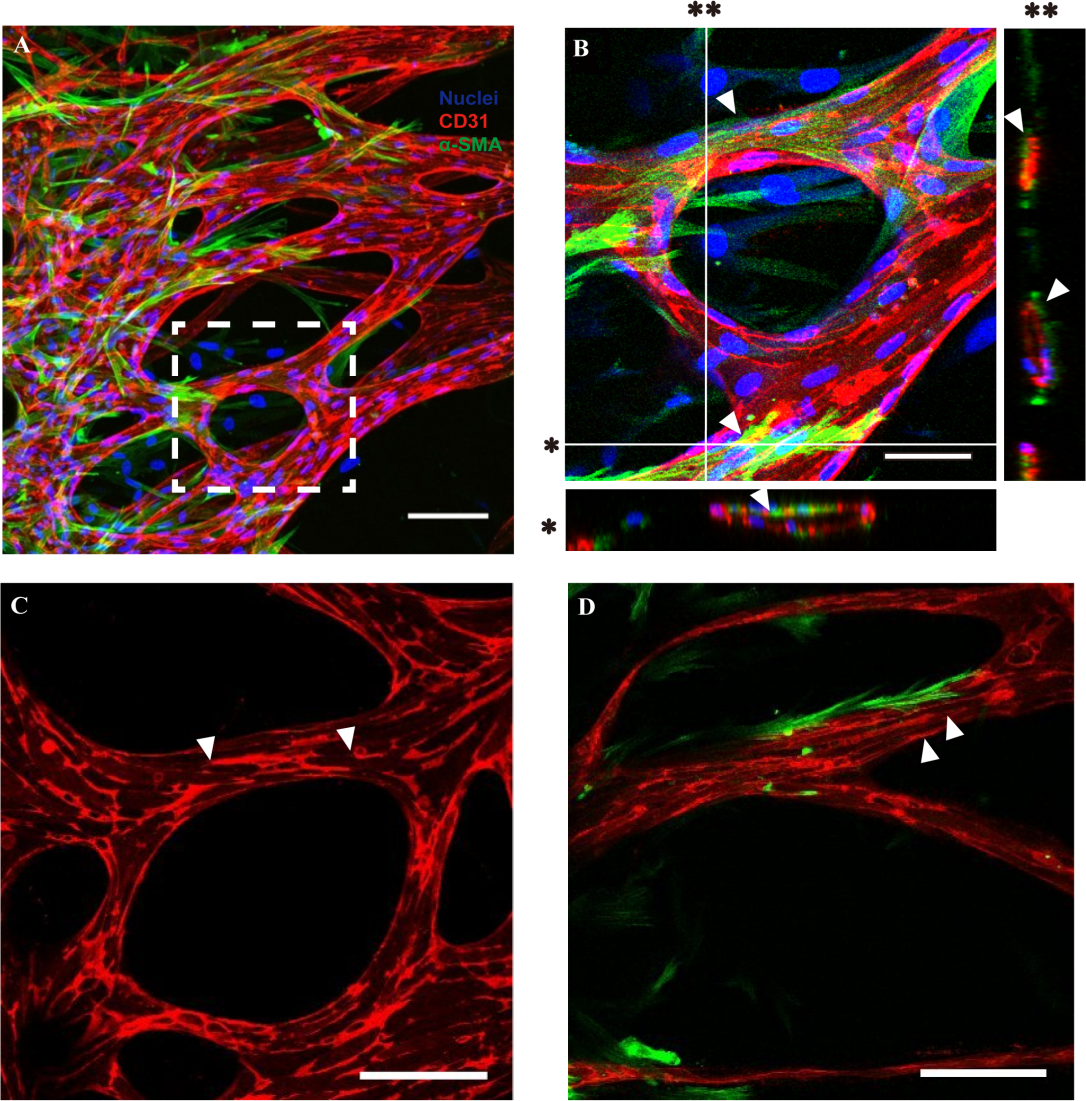
Morphology of pericytes covering the abluminal surface of the blood vessel and their effect on PECAM-1 expression. (A) The confocal micrograph shows that pericytes are evenly distributed on the blood vessel network while covering the vessel. (B) Intact covering of the vessel with pericyte processes, as well as lumen formation of the blood vessel, can be observed based on the enlarged image of the white dotted box in (A). (C, D) PECAM-1 expression is broken occasionally in EC-only blood vessels (C) compared with continuous expression in pericyte-covered vessels. Microvascular networks were stained with anti-CD31 (red), the nucleus with Hoechst 33342 (blue), and pericytes with α-SMA (green). Scale bars, 100 μm (A, C, D) or 50 μm (B)

We first verified the EC-pericyte association of the vascular network generated by angiogenic processes within our microfluidic platform. Whole vascular networks covered by pericytes, especially on the abluminal endothelial surface (not in the acellular fibrin matrix), could be confirmed through 3D images obtained by confocal microscopy (Fig 2A). The intact coverage of pericytes and lumen formation within the vascular network was clearly identified based on cross-sectional views of the magnified image (Fig 2B). Notably, longitudinal process formation of pericytes could be observed on the vessel-enveloping pericytes, recapitulating the morphological characteristics of blood vessel-enveloping pericytes *in vivo* (Fig 2B arrowheads) [26]. Furthermore, platelet endothelial cell adhesion molecule-1 (PECAM-1), the adhesive protein expressed at intercellular contacts in the endothelium, was expressed more evenly and clearly when co-cultured with pericytes (Fig 2C and 2D, arrowheads).

**Fig 3:**
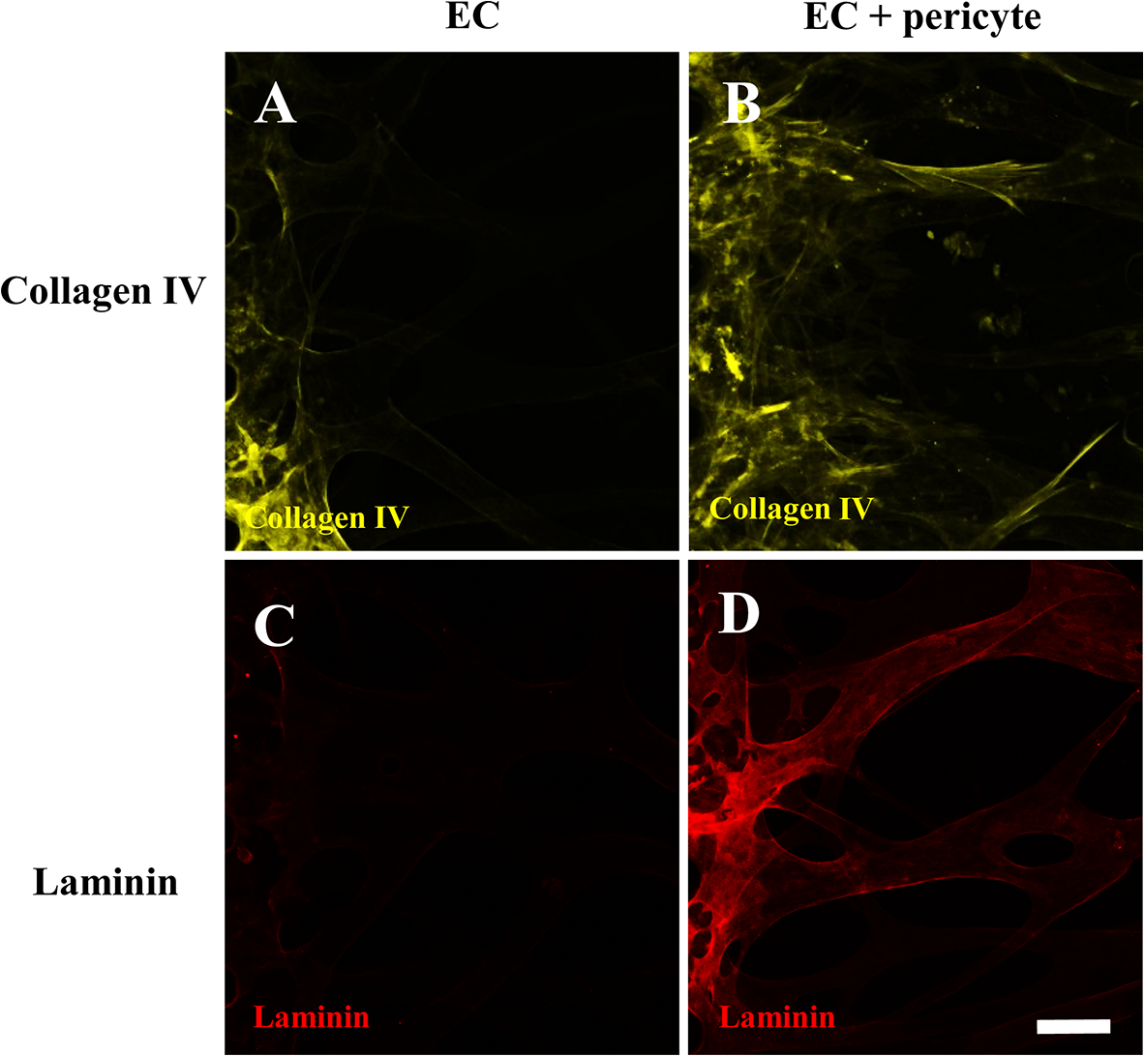
Basement membrane deposition of EC and EC-pericyte conditions. Immunofluorescent images show collagen IV (A, B) and laminin (C, D) deposition along the vascular abluminal surfaces. The fluorescent intensity of the basement membrane is significantly higher in pericyte-covered blood vessels (B, D) compared with EC-only vessels when stained and imaged under the same conditions. Scale bar, 100 μm.

Basement membrane assembly on the abluminal surface of blood vessels is required to intimate the interaction between ECs and pericytes as a critical step of vessel maturation *in vitro* and *in vivo* [15]. Thus, we explored the effect of pericyte co-culture on the basement membrane assembly around the blood vessel network. Interestingly, fluorescence staining of laminin and collagen IV, the major components of the basal lamina, showed sharp increments in the composition around the vessel network in pericyte co-cultures (Fig 3B and 3D) compared with EC-only vessels (Fig 3A and 3C). This suggests that our model represents maturation of the vascular network through EC-pericyte interactions *in vitro* [2, 27]. Taken together, pericytes in our platform not only showed well-differentiated morphology but also played an important role in maturation of the newly formed blood vessel network.

### Pericytes regulate the morphology of the sprouting EC network

**Fig 4:**
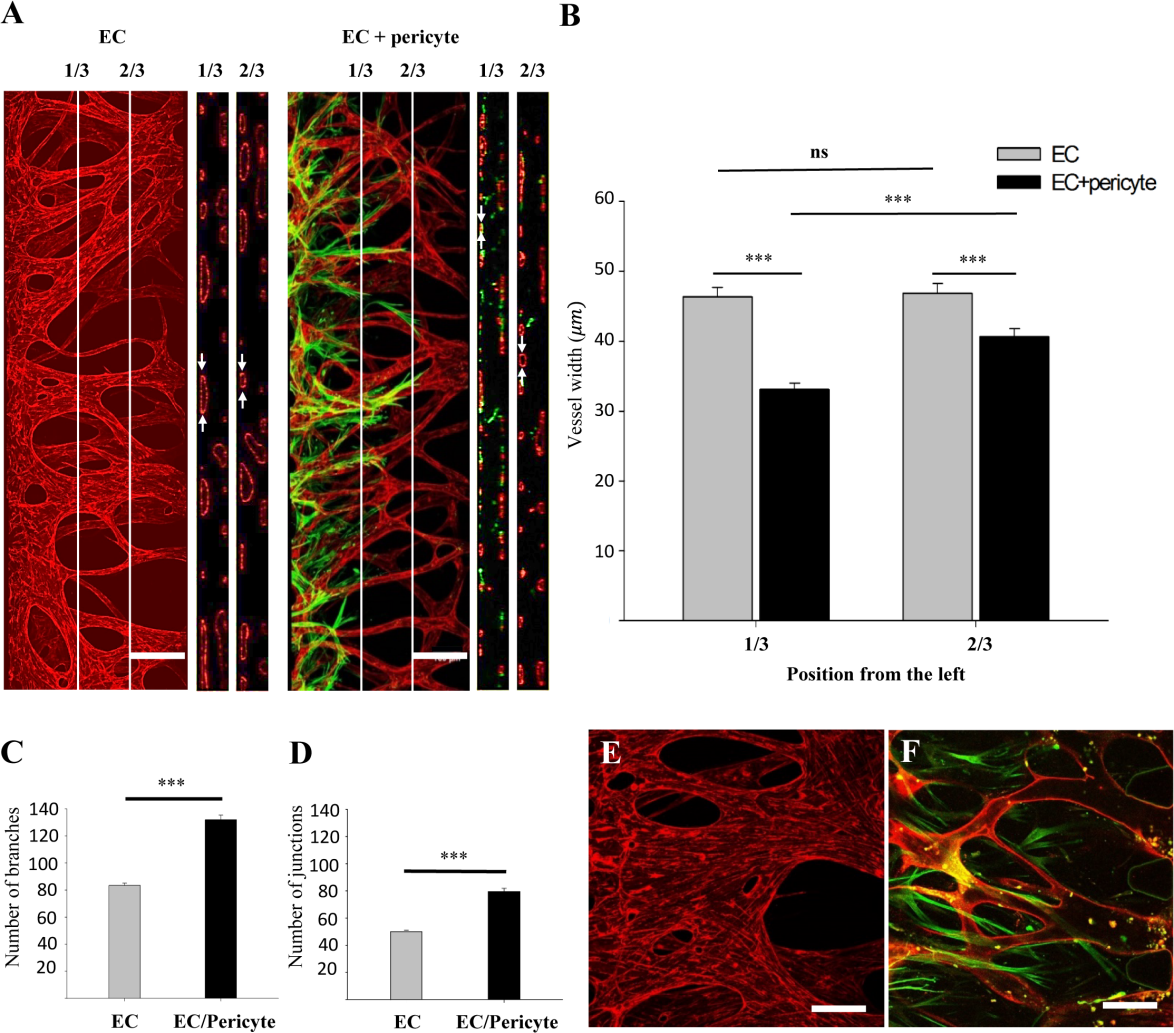
Characterization of vascular morphological properties and comparison of EC monocultures and EC-pericyte co-cultures. (A) The 3D confocal image sectioned along the lines that divide the width of ROI into three equal parts. Cross sectional view of each portion clearly shows lumen formation of the blood vessel network (white arrows). (B) Vessel widths were measured along the sectioned lines. When two cells were cultured together, vessel width decreased significantly. The width of the pericyte-covered vessel increased again at the point region. (C, D) The numbers of junctions and branches increased significantly under co-culture conditions. The protective effect of pericytes from vessel dilation was significant when compared with the EC-only vessel at day 7 (E) and the pericyte-covered vessel at day 11 (F). CD31 (red) shows staining of ECs and α-SMA (green) shows staining of pericytes. Scale bar, 200 μm (A, B) or 100 μm (E, F) (n = 28 for EC monocultures, n = 26 for EC-pericyte co-cultures. *** p < 0.001).

EC hyperplasia in blood vessels lacking pericytes has been reported previously [5]. We have also observed rapid and continuous dilation of the EC network formed on microfluidic devices [12]. Although the retarding effect of pericytes on vessel dilation processes was clear to the naked eye (Fig 4A), we performed computational image analysis to determine quantitatively which properties of the vessel network were affected by pericyte coverage. The 3D confocal images of each sample was sectioned at 1/3 and 2/3 points, and the diameter of each vessel was measured. As expected, the vessel network covered by pericytes showed significantly narrower vessel diameters compared with the network comprised of ECs only (Fig 4B). Interestingly, under EC-pericyte co-culture conditions, the vessel diameter measured at 1/3 points was narrower than that at 2/3 points, most likely due to the abundance of pericytes near the EC-pericyte-attached hydrogel wall. This indicated that the vessel regulatory effect was confined to the proximal regions of the pericytes themselves. Moreover, EC-pericyte co-cultures showed intricate network morphology in terms of the number of branches (79.6±2.15 to 131.9±1.84) and junctions (50.1±1.14 to 83.54±0.86) (Fig 4C and 4D). The regulatory effect of pericytes on blood vessel dilation was even more drastic when the samples were cultured for longer periods. EC-only vessels were dilated severely and lost their network shape after 7 days of culture (Fig 4E), while the pericyte-covered vessel network maintained its morphology over 10 days (Fig 4F).

### EC-pericyte association decreased EC permeability

**Fig 5:**
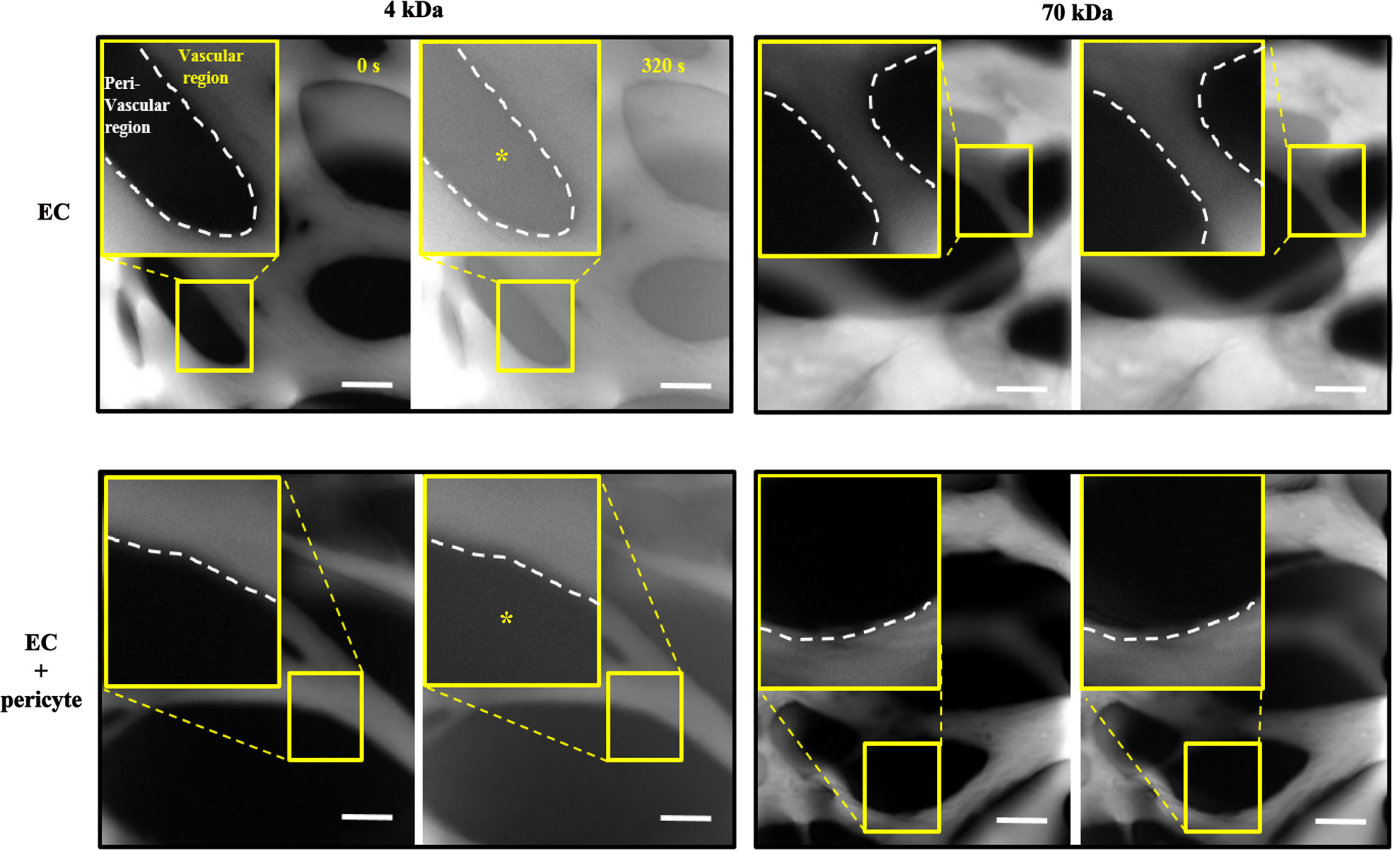
Permeability difference between EC monoculture and EC-pericyte co-culture conditions. FITC-dextran of two different molecular weights was introduced into the permeable vascular network. EC-only vessels showed significant leakage of 4 kDa FITC-dextran compared with pericyte-covered vessels (left panels). No leakage difference was observed with 70 kDa FITC-dextran (right panels). The white dotted line indicates the boundary between the vascular and peri-vascular regions. Scale bar, 100 μm.

Permeability measurements are commonly used for direct visualization of blood vessel barrier function. We have shown that the engineered blood vessels generated by natural morphogenic processes showed much lower permeability compared with the other *in vitro* blood vessel models [25]. Several studies have described increased permeability under pericyte-dissociated conditions [28]. Thus, we explored whether the association with pericytes could improve functionality of the engineered blood vessel network in terms of permeability. FITC-dextran molecules with molecular weights of 4 kDa and 70 kDa were introduced into the openings of the vascular network. Fluorescent images taken at 0 and 320 seconds were compared to clearly show the gradual permeation of dextran from the vascular into the peri-vascular region (Fig 5).

There was no significant leakage of 70-kDa FITC-dextran over 320 seconds in either the EC-only or EC-pericyte vascular network (Fig 5, right panels). However, 4 kDa dextran leaked out much slower in the EC-pericyte co-cultured vascular network compared with the EC-only vascular network, indicating that a decrease in permeability was associated with the pericyte co-cultures (Fig 5, left panels).

### Modulation of EC-pericyte interaction with angiogenic factor and inflammatory cytokines

To demonstrate a potential of our platform as a drug evaluation tool, we tested the effect of various biochemical factors on EC-pericyte association and angiogenesis. VEGF-A, TNF-α and IL-1α were tested, all of which are known as key molecules that regulate angiogenesis via various mechanisms, especially in the tumor microenvironment [29].

**Fig 6:**
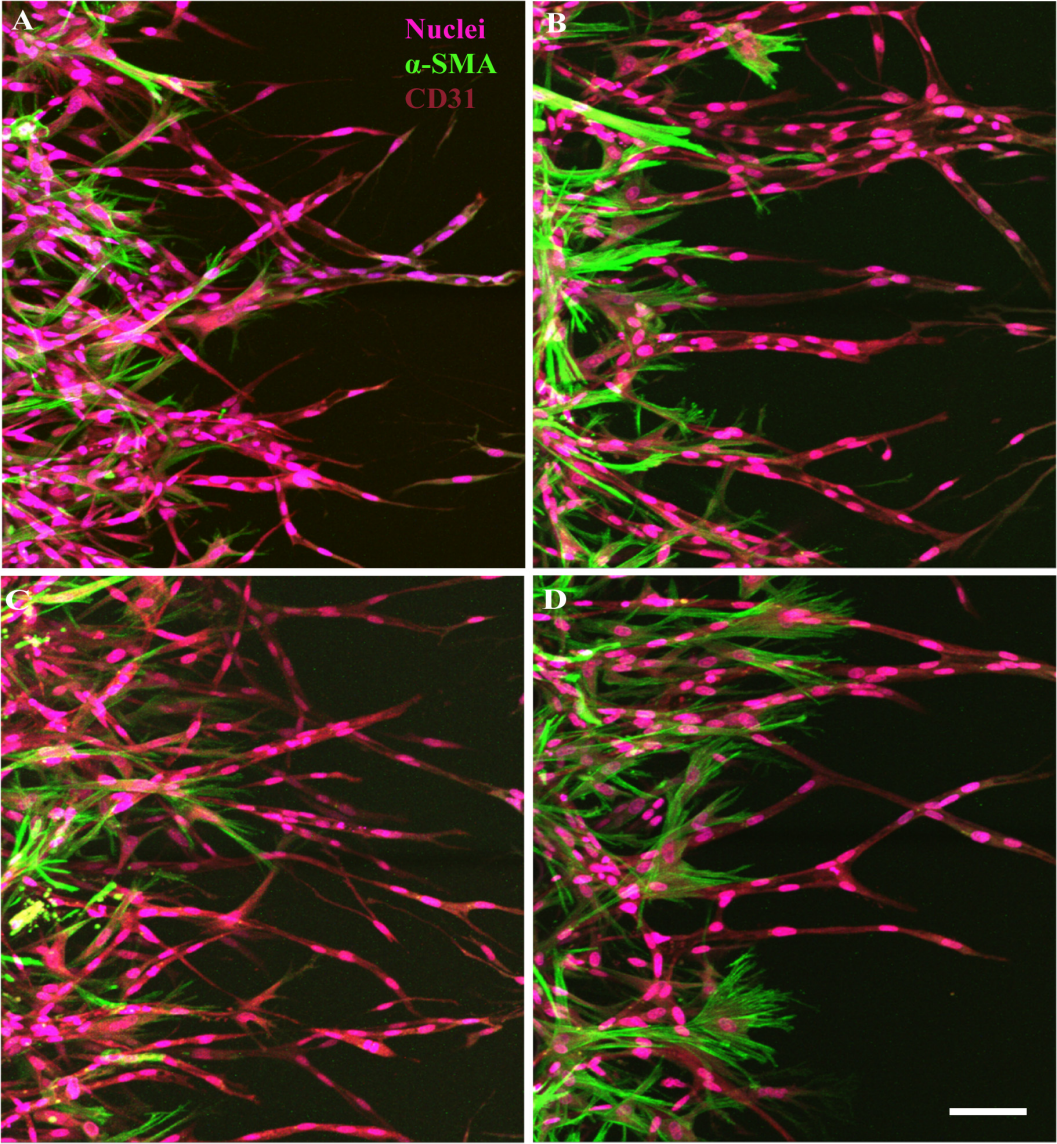
Effect of VEGF-A, TNFα and IL-1α on EC-pericyte association. Representative confocal images showed contrasting morphology of the blood vessels in response to different biochemical factors. In comparison with the control condition (A), VEGF-A (100ng/ml) treated vessels were dilated and pericytes showed contracted morphology (B). Inflammatory cytokines TNFα (10 ng/ml) (C) and IL-1α (10 ng/ml) (D) treated pericytes showed both distinct filopodia growth, but in different morphology. Scale bar 50 μm.

Not only ECs sprouts but pericytes exhibited distinct morphology when grown under VEGF and inflammatory cytokines. In particular, VEGF treatment exhibited thick and dilated vessels as expected (Fig 6B). Interestingly, VEGF repressed an extension of the pericytes, whereas inflammatory cytokines stimulated them to project significantly more filopodia. Although both inflammatory cytokines showed similar effect on the pericytes in the aspect of filopodia projection (Fig 6C and 6D), the morphology of ECs and pericytes were clearly be distinguished in each cytokine condition. TNF-α made the ECs to sprout thin branches, while IL-1α exhibited less or negligible differences in ECs. Morphology of pericytes was also different in the two conditions, since pericytes treated with TNF-α showed extended and branched filopodia, while pericytes treated with IL-1α showed thinner and short, but much more filopodia from the leading edge of the cell body.

Taken together, these results showed close agreement with previous reports that discussed about the molecular interaction between ECs and pericytes *in vivo* [30–33], thus are demonstrating the physiological relevancy as well as availability as an advanced drug evaluation platform of our system.

## Discussion and Conclusions

In the majority of previous reports, pericyte-covered blood vessels have been generated *in vitro* using mixed cultures of pericytes and ECs embedded together in a 3D hydrogel. Although experimentally simple, this method could not mimic the sequential steps of angiogenic processes or produce perfused blood vessels [15–18, 22, 24, 34]. Unlike previous studies, we aimed to mimic the sequential steps of neovessel formation—sprouting, elongation and maturation—by culturing pericytes together with ECs.

Since LFs induce angiogenic sprouting from the EC monolayer in various *in vitro* microvascular systems [25, 35], we first used LFs to induce angiogenesis in our EC-pericyte co-culture system. Although LFs were a good initiator of neovessel formation, they failed to form a physical association between ECs and pericytes during co-culture (S1A Fig). It is inferred that LFs secrete excessive pro-angiogenic factors such as VEGF-A which is unnecessary during vessel maturation [36] and interfere EC-pericyte interaction [30].

Angiogenic sprouting from EC-pericyte co-cultures could embody the sequential steps involving pericyte association around blood vessels, as well as perfusion of the vessels [4, 37]. Specifically, the immature, poorly lumenized initial EC sprouts that migrate ahead of the pericytes were finally perfused and wrapped by the pericytes (S1B Fig). We also demonstrated that pericytes prevent enlargement of blood vessels, thereby stabilizing (Fig 4F) and helping ECs to establish a denser vascular network with more interconnecting junctions and branches (Fig 4B), resembling the vascular developmental procedure under physiological conditions. These results were in good agreement with previous reports [17, 23], supporting the fact that pericytes play a role in the regulation of vessel diameter and branching [5, 36].

We also show a sharp contrast in permeability between EC-only and EC-pericyte blood vessels using 4 kDa FITC-dextran (Fig 5). Unfortunately, the results could not be shown in a quantitative manner, since the platform was designed for morphological observation, rather than quantitative analysis of permeability. In this platform, many gaps between the posts allowed us to observe *in vivo*-like angiogenic sprouting as well as robust network formation. However, those gaps caused inflow of the fluorescent dyes direct into the perivascular ECM region rather than through the EC barrier, thus contaminating results of image-based permeability measurement. We have developed the newly designed platform to overcome this limitation [25]. Since permeability is a key character to represent functionality of blood vessel, further studies are required to develop an in vitro platform that enables measurement of barrier function in a quantitative manner without losing morphological characteristics of blood vessel.

Despite the importance of EC-pericyte interaction in many diseases, to the best of our knowledge, yet no *in vitro* platform enables evaluation of biochemical factors on EC-pericyte interaction under the angiogenic condition. Notably, it is known that aberration of EC-pericyte cell signalling in the tumor microenvironment initiate angiogenesis, thus targeting them is one of the candidates in anti-angiogencic drug development [38–40]. In order to demonstrate the potential of our platform as an *in vitro* model of the EC-pericyte biology especially under the tumor microenvironment, we tested the effect of VEGF and inflammatory cytokines on EC-pericyte association in the system. Endothelial cells and pericytes both showed distinct morphological changes in response to those biochemical factors. Specifically, the pericytes showed constricted features under excessive VEGF-A. The result is in close agreement with those of previous reports that proved VEGF-R2 signalling pathway restricted proliferation and migration of the vascular smooth muscle cells *in vitro* and *in vivo* [30]. By contrast, the pericytes exposed to inflammatory cytokines exhibited invasive morphology with increased filopodia. The results not only corresponded to the previous reports on the effect of TNF-α [33] or IL-1α [31, 32], but also demonstrated the advantages of our platform for detailed observation of the cells.

Taken together, we developed advanced engineered blood vessels with *in vivo* similarity and morphology by co-culturing ECs with perivascular cells. We expect that our microfluidic model of pericyte-enveloped blood vessels enables us to have better understanding of vascular diseases owing to the malfunction of pericytes by allowing dynamic observation and manipulation of pericytes in a perfusable vessel network.

## Supporting Information

S1 Fig Differences in appearance of EC-pericyte coverage depending on the fibroblast type. Whole device image of the microvascular networks induced by (A) lung fibroblasts and (B) dermal fibroblasts. (C) Cross-sectional plane of the yellow dotted box in (A) shows poorly attached pericytes on the vessel network (white arrowheads). (D) Cross-sectional plane of the yellow dotted box in (B) shows that most of the pericytes were well adjoined to the vessel network (white arrows). Endothelial cells and pericytes were stained with anti-CD31 (red) and anti-α-SMA (green), respectively. Scale bars, 100 μm.
(TIF)
