# Supplementary figures and images for: Engineering of a Biomimetic Pericyte-Covered 3D Microvascular Network
Source: PLoS One. 2015 Jul 23;10(7):e0133880. doi: 10.1371/journal.pone.0133880 (PMC4512698; doi:10.1371/journal.pone.0133880)

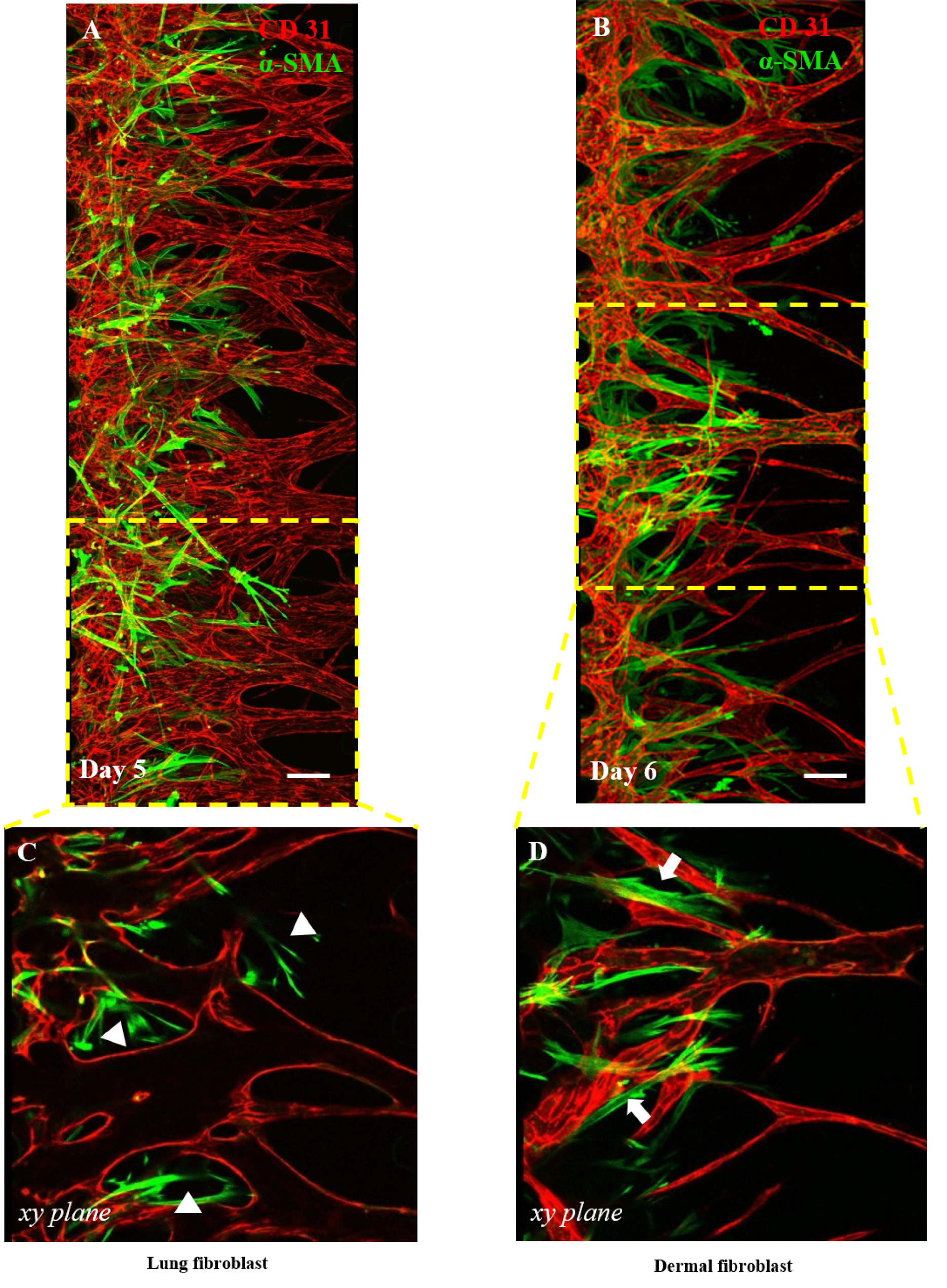

Supplement: S1 Fig — Whole device image of the microvascular networks induced by (A) lung fibroblasts and (B) dermal fibroblasts. (C) Cross-sectional plane of the yellow dotted box in (A) shows poorly attached pericytes on the vessel network (white arrowheads). (D) Cross-sectional plane of the yellow dotted box in (B) shows that most of the pericytes were well adjoined to the vessel network (white arrows). Endothelial cells and pericytes were stained with anti-CD31 (red) and anti-α-SMA (green), respectively. Scale bars, 100 μm. (TIF) [file pone.0133880.s001.tif]
